# On Hallucinations in Insanity

**Published:** 1862-07

**Authors:** A. Brierre de Boismont


### Art. IX.—ON HALLUCINATIONS IN INSANITY.

By A. Brierre de Boismont.

(*Concluded from page* 298.)

### III. *Of Hallucinations and Illusions in Melancholic Monomania (Lypemania).*

The hallucinations and illusions of melancholic monomania (lypemania of Esquirol) are characterized by fixity, intensity, and tenacity, as well as by expression, which distinguish them from those observed in acute delirium and mania. The integrity of the reasoning power, excepting always some obscured portions, permits us in most cases to analyse the phenomena and follow their modifications with great minuteness. The patient himself, when he is intelligent, may furnish very useful indications upon the return of his conceptions as sensations. Although in acute delirium and mania the false sensations are often painful and distressing, the nature of the intellectual disorder removes from the ideas and their sensible signs, which are their forced reflexes, that morbid fixity which is the pathognomic characteristic of melancholic monomania. In the latter case, the affliction possesses a hopeless immobility, and what surprises the observer is that catastrophes are not more numerous.

In 303 patients suffering from this variety of madness, we counted 248 who experienced hallucinations and illusions, and of this number 212 presented all the expressions of grief.

The following table shows the distribution of these morbid sensorial perceptions, which we classify in the first instance under three categories, according as they are isolated or united, and which are arranged under each division according to numerical order.

FIRST CATEGORY.

*Hallucinations alone.*

| | |
|---|---|
| Of Hearing | 55 |
| Of Hearing and Sight | 31 |
| Of Sight | 7 |
| Of Hearing and Touch | 1 |
| Of Sight and Touch | 1 |
| | 95 |

SECOND CATEGORY.

*Hallucinations and Illusions united.*

| | |
|---|---|
| Hallucination of Hearing and Illusions of Hearing, of Sight, of Touch, of Smell, of Taste, of General Internal Sensibility (Hallucination associated with 1, 2, 3, 4 Illusions) | 51 |
| Hallucinations of Hearing and Sight, and Illusions of Hearing and Sight (united), of Hearing and Sight (separate), of Touch, Smell, and General Internal Sensibility (subject to same remark as above) | 41 |
| Hallucinations and Illusions of all the Senses | 19 |
| Hallucination of Sight and Illusions of Hearing, Sight, Touch, Smell, Taste, and General Internal Sensibility (subject to same remark) | 11 |
| Hallucination of Touch and Illusion of Touch | 1 |
| | 123 |

THIRD CATEGORY.

*Illusions alone.*

| | |
|---|---|
| Of *Sight alone*, or in association with Illusions of Touch, Smell, Taste, and General Internal Sensibility | 11 |
| Of *Hearing alone*, or in association with Illusions of Sight and Taste | 8 |
| Of *Touch alone*, or in association with Illusions of Smell and Taste | 3 |
| Of *Smell alone*, or in association with Illusion of Taste | 4 |
| Of *Taste alone*, or in association with Illusion of Sight | 3 |
| Of *General Internal Sensibility* | 1 |
| | 30 |

*General Summary.*

| | |
|---|---|
| Hallucinations alone | 95 |
| Hallucinations and Illusions united | 123 |
| Illusions alone | 30 |
| | 248 |

These various combinations are those which we have actually observed, but it is evident that many others are possible. We may also remark, that although hallucinations of touch, smell, and taste are very rare or appear wanting in our observations, this is because we have almost always found with regard to morbid perceptions of these senses that there existed a real sensation transformed by illusion. In this there is nothing surprising when we consider the great number of changes which persons and things undergo with the insane. There are evidently many hallucinations of touch, smell, and taste, and we have reported several such cases in our second edition on *Hallucinations*, pp. 105 et seq., but observation establishes the fact that the illusions of these three senses are much less frequent.

### First Category.—*Hallucinations alone.*

In studying the analysis of the sensorial expressions which characterize the hallucinations and illusions of melancholic monomania, we shall pursue the same order which we adopted with regard to mania, that is to say, their numerical proportion in each species of hallucination and illusion.

If uniformity of expression determine a type, melancholic monomania should be most constant. We have already noticed the frequency of painful and distressing sensations in acute delirium and mania; in lypemania they are, so to speak, stereotyped. We have observed them in 53 out of 55 cases of hallucination of hearing.

Melancholic monomaniacs, who are a prey to hallucinations of hearing, hear disagreeable, offensive, painful, and scoffing remarks: the voices mock them, speak of them as subjects of ridicule or cowards, tell them that they are fools or that people pass them as such, and in other circumstances they murmur complaints and words of sadness which increase their grief.

The most numerous voices, and those which are the torment of the insane, proceed from enemies and persecutors. Professor Lasegue has called attention to this predominant variety of delirium in lypemania;* but he has connected it in his reports rather with delirious conceptions than false sensations; it may be said that these form three-fourths of the hallucinations and illusions of lypemania.

The voices pursue the patients with unfounded reproaches, and overwhelm them with taunts and threats. They tell the patients they are ruined, lost, and dishonoured. The threats are

* Ch. Lasegue, Du Délire de Persécution, *Archives Générales de Médecine*, t. iv. 2nd series, p. 435.

sometimes the echoes of real events. Thus, one of these patients had lost a considerable sum of money, and in his case the voice was spoken thought. Oftener than we think, hallucination and delirious conception are manifestations and exaggerations of physical or moral fact.

The threats uttered by the voices may assume greater importance according to the patient's degree of mental organization, impressionability, or education. They tell some that they are sought for, that some one wishes to harm them, or send them to prison. Some are accused of theft and every species of crime. They are about to be arrested, sent to prison, and condemned; the police are below waiting for them. The first patient we attended, rendered desperate by such threats, drowned himself in a water-butt.

The voices often describe frightful tortures as reserved for the patient; he is to be put in the pillory, or into a cave, to be flayed alive, cut in pieces, killed, or guillotined.

To others the voices announced that they are poisoned, that all their relations and friends have been killed; this hallucination has been observed in patients whose friends have not visited them for some time, and it is necessary to be careful of prolonging isolation when not required for treatment. There are others to whom the voices declare they have committed great sins, that they are damned, delivered over to the devil and the flames of hell, or that the fire of heaven is about to fall on them, or on the house in which they are.

Ordinarily these voices plunge the patients into a state of unspeakable terror and despair, and it then becomes necessary to have recourse to measures to prevent them committing suicide by hunger or otherwise. We have often heard some of these patients ask leave to write to the Emperor to implore him to deliver them from their persecutors. This is one of the many examples which prove that in our country man relies more on authority than on himself.

The hallucinations of sight alone, which are much less numerous, according to our observation, than those of hearing, being represented by only seven cases, consisted of threatening figures, sometimes confused, sometimes distinct. One man saw his cows which thieves wished to steal, and he refused to go to bed in order that he might protect them. One lady desired her sister to take away her children from her side for fear she should crush them. In other cases the objects were only perceived through mist or obscurity; the patients could not describe them; they only said they saw figures without entering into any explanation. Sometimes the apparitions were entire, at others they saw only a part. One young lady said there was an eye continually before her.

Out of seven hallucinations of sight six were of a painful nature.

The hallucinations of hearing and sight, in number 31, were characterized in 24 cases by painful impressions. The patients believed that they were surrounded by people who wished to do them harm. They perceived malefactors, who entered by the window or walked on the roofs, assassins who wished to kill them. "Don't you see them?" said these patients; "they are attacking us—they will kill us." Nothing could preserve them; the thieves had entered by the chimney or passed through the wall. The impossible and the absurd has no existence for the insane.

In general the apparitions were hideous and frightful figures. With ignorant and weak-minded persons visions of sorcerers and of the devil coming out of hell are common. The latter variety is especially observed in women.

In two cases, hallucinations of hearing and sight existed with hallucination of touch. One individual received blows on the head, the back, and the feet; another affirmed that when any one knocked at his door he felt a corresponding blow on his body—a fact observed in nervous persons.

As with delirious conceptions, hallucinations are the reflex of the dominant ideas of the period. In analysing the 1146 observations of our establishment, and especially the 861 made by ourselves since the year 1848, we were able to verify this remark during the progress of the events of the Revolution of February, and after the terrible conflicts of June.

The following is what we then wrote on this subject in the *Union Médicale* (20 July, 1848), p. 335:—

"Scarcely were the last guns fired when I received in my two establishments several victims of this revolution, which, as was well remarked, has come about much too rapidly. In general, these first patients were sad, cast down, melancholic (lypemaniacs); they believed that they were in danger of being massacred. One of them, a man of great learning and author of several esteemed works, would sit immovable, his eye fixed, and scarcely spoke a word; he was convinced that he was about to be thrown down a sewer, to be there suffocated, and this horrible threat resounded incessantly in his ears. Another cried out each instant, 'There they are, they are breaking open the door; they are coming to take me, they are going to shoot me.' Many heard voices which threatened that they and their relations were about to be guillotined, or they heard continual discharges of firearms. A great number of these patients belonged to the burgess class—persons who, by dint of labour, intelligence, and perseverance, had made for themselves a position which many desire to have without this trouble. In order to escape these threatened disasters, many of these unfortunates attempted self-destruction, and it required constant watching to prevent them putting their sinister projects into execution. Several of them perceiving they were never lost sight of, resolved to die of hunger. One would have been at a loss to conceive of the savage energy displayed by the patients of this class. Out of six of them who imagined themselves to be great criminals, or ruined, or denounced by their neighbours, two succumbed, notwithstanding the use of the stomach-pump. One of the latter presented a singular illusion, which we have met with several times since: he was persuaded that his œsophagus had been stopped up, and that all passage for food was closed. He would cry—'How can you expect a man to live when you introduce food by the respiratory passages? You choke me; I shall soon be dead.'

"It was not long before I observed a fresh series of patients, which might have been called the evidence of new ideas. These no longer walked with abased head and sorrowing eye; their aspect was proud, joyous, and exalted; they talked incessantly, wrote memoirs and constitutions, proclaimed themselves great personages, the saviours of their country; they were generals, members of the executive, and their hallucinations partook of their mental disposition.

"The days of June came. The patients of this second epoch presented several cases of furious madness. Those who were attacked wished to kill, massacre, and shoot all the world; they groaned and cried out: 'To the guard-house! Assassinate him!' Their hallucinations reproduced the noises and the emotions of combat. They were in a state of exultation which it is impossible to describe.

"The greater number of these patients were melancholic monomaniacs. Like those of the corresponding category of February, they spoke of death, the guillotine, ruin, pillage, and rape. The frightful scenes which had taken place under their eyes had plunged them into a sort of stupor. One lady said, 'Before this frightful revolution I was of a happy disposition; but how can one preserve one's reason in the midst of constant fear for the life and fortune of those we love, with the certainty of losing all we prize? It is these events which have brought me to the state I am in. I feel extreme terror; I shudder at the slightest movement—at the gentlest sound; I try to reason with myself, but I have no longer the strength to do it.'"

Although twelve years have elapsed since these lines appeared, we need not modify them; we might even add to what we then stated, for we still continue to receive patients whose disease dates from those deplorable dissensions; but what has just been read suffices to establish, not merely the influence of dominant ideas on the kind of delirium, but also the extreme predominance of painful impressions in lypemania.

SECOND CATEGORY.—*Hallucinations associated with Illusions.*

The second category which we are about to pass in review comprehends 123 cases of hallucination associated with illusions, of which 103 are exclusively of a painful nature; and amongst them we shall again meet with false sensations of enemies, persecutions, poison, theft, crime, threats, slanders, arrests, convictions, punishments, death, ruin, dishonour, devils, hell, mockery, grimaces, change of figures, bad smell and infected taste, blows, violence, and immodest actions, &c. The hallucinations, however, are more intensified, because they are composed of elements both more numerous and persistent.

In fact, the hallucinations no longer manifest themselves singly or in pairs; they combine with illusions so as to form binary, ternary, and quaternary, and often general associations, since we have met with all forms united in 19 cases. The patient in such cases lives in a world of shadows which disturb his ideas, displace the whole of reason, advice, and consolation, considerably aggravate his condition, and reduce him to a state of despair. One of the illusions, the frequency of which we have already remarked, causes him to believe that he is the victim of an endless joke which is being played off against him, and in which relations, friends, patients, and servants have each their parts. One lady subject to hallucinations imagined that the boarders who were tranquil or in a way of recovery were sham fools, and was most angry with them, while she reserved all her affection for the real fools. To the subjects of hallucination nothing is real, all appearances are false and deceitful; theirs is the true world of Berkeley.

We shall not return to the painful symptoms which we have already considered, but will merely indicate the shades and modifications which were not presented to our notice under the first category, and we shall add a few words respecting some false sensations which appear in a more marked form in cases belonging to this class. Where hallucinations were associated with illusions, the changes of persons and objects were very frequent: we have observed this feature in forty cases. One of these patients entered into the most circumstantial details respecting the machinations of his enemies, their words and actions, and he reckoned amongst them relations, fellow-boarders, and domestics; his was really a conclusive example of that which some years ago obtained the name of systematic madness (*folie systématique*). It would be difficult to conceive of the subtilty and obstinacy evinced by these patients in interpreting, according to the requirements of their hallucinations, all the sensations which they experience. One lady bewailed that she was the object of public ridicule; that she was treated as an idiot and a fool. If any one laughed or expectorated, it was to scorn and insult her. Whatever she heard outside possessed a malevolent signification towards her: the street cries, the barking of a dog, the neighing of a horse, the cracking of a whip, the clinking of broken bottles, all suggested the idea that some one was incriminating her words or her thoughts. She resolved that she would neither speak, nor see, nor hear. To all objections she found an answer, and her arguments were often embarrassing. This system of interpretation never recoils in face of a difficulty. A young lady when out walking came across a mason's scaffolding, which she persuaded herself was the instrument of her punishment: it was vainly attempted to demonstrate to her the falsity of her impression, she obstinately persisted in her idea, and returned in a state of profound grief.

Amongst the facts relating to persecutions, enemies, and interpretation, the following suggests more than one lesson :—

On the 30th December, 1839, M. D. was received into my establishment from Bicêtre, where he had been placed two months before for an act of madness. He was the son of a merchant who counted his riches by millions, and having himself tasted the enjoyments of wealth, he had seen his long flourishing fortune crumble away in consequence of an uninterrupted series of catastrophes. Being obliged to depend upon tuition for a livelihood, and often reduced to the strictest necessaries of life, his privations exercised a melancholy influence over his ideas; irresolution, dejection, and despair were the unhappy results; thence to madness was but one step, and it was soon accomplished.

When he came before me I found him timorous, and alarmed at the slightest question. He complained of being cold, which is not unfrequent with lypemaniacs; but what troubled him most was another thing, viz., hearing enemies speaking to him through the walls, being harassed by individuals who put objects of value in his pillow-case or his mattress with a view to make him out a thief and to dishonour him. This idea did not leave him a moment's repose. He passed his days in groaning and lamenting that he was about to suffer the most cruel torments. He was in vain reminded that he had been repeating the same thing for ten days and yet nothing had happened, and in vain was he surrounded with attention and evidences of good-will; he was insensible to all. I do not know a more afflicting spectacle than that of a melancholic patient arrived at this stage of the disease, and after having witnessed it several times, I understand the contagion of example and the temptation to suicide.

In order to lessen his distress, I directed his pillow-case to be unsewn, which he pretended the malice of his enemies had caused to be filled with diamonds, although he never was able to tell us why they had conspired to ruin him. After having examined the contents with the greatest attention, he was tranquil for the remainder of the day ; but the next day his ideas were the same, and when we proposed to repeat the experiment, he replied to me, in a despairing tone, that his unseen enemies had already taken care to remove the diamonds.

This unhappy man was worse at night than during the day. At one time he saw a man come and take his clothes, in order to fill his pockets with precious stones. At other times his persecutors met in greater number, put him in a bath, and beat and otherwise ill-treated him ; in the morning he affirmed that his body was bruised all over in consequence of what they had done to him, or that they had taken him up and transported him to different towns in France, Algeria, or America. The descriptions he gave of those places were confused ; often he did no more than name them. His plate, the wall, the curtains, appeared to him to be filled with people and ships come to carry him away. When he was served with food, he never took the portion intended for him, but that of another person, because at last he came to the conclusion that we wished to poison him. He wiped each plate set before him with a handkerchief to remove the particles of poison. His beverages caused him horrible suffering on account of their venomous odours, and he never emptied his glass, supposing that the poison remained at the bottom. One of his great troubles was to be left alone in an apartment where there was silver plate, being apprehensive that he might be accused of theft.

This same man, whose false ideas were unconquerable, took part in general conversation with remarkable aptness, from the moment that his attention was fixed by any unforeseen thing.

As the despair which was occasioned by his permanent idea of theft refused to yield to any moral treatment, we feared that he might have recourse to suicide, and he therefore was subject to constant surveillance. It not unusually happens with patients who fancy that some one wishes to poison them, that the impressions caused by food are such as to lead to a daily diminution of the quantity taken. This progressive abstinence results in dyspeptic symptoms, which go on increasing until the patients swallow with the greatest difficulty the small quantities of food which one is able to make them take. Several complain of acute pains in the pharynx, the œsophagus, or the stomach, and deglutition becomes distressing.

M. D., who for some time had eaten very little, commenced to maintain that some one put copper and sponge into his throat and stomach. He did not conceal from us that he thought my wife, my children, and I were in concert to poison him. He asked pardon for entertaining such an idea, admitted that it must appear strange, and that any one but himself who should hold such language would justly be treated as a fool; but he affirmed that what he stated was really true.

The life of man is but a long series of contradictions; in the twinkling of an eye he passes from black to white, and says and does the contrary of what he said and did a minute previously. The actions of the insane are but the exaggeration of this singular defect. Here, for example, is an unhappy man who, in terror at the thought of dying by poison, condemns himself to all the horrors of hunger, submits to a real suffering, in order to escape imaginary torments; who fears death, and yet kills himself. In vain does he see persons who dine with him eat the same meats and drink the same wine; nothing calms him; and he persists, notwithstanding, in thinking that the head of the establishment, who has the greatest interest in preserving his patients, does all in his power to poison them without being able to assign a motive. Can it be true, as moralists have affirmed, that the ills of the future will be worse than those of the present?

Four months after his arrival, an extreme emaciation announced the serious attack made upon his organization by this regimen; the pulse was feeble and slow, the skin was remarkably cold, particularly at the extremities, the face was of an unearthly yellow complexion; for some days he had had a slight dry cough, his breath was insupportably fœtid. Shortly after, the progress of the disease brought on, first a hoarseness, and then the total loss of his voice: it became necessary to approach him very close, in order to catch a word here and there.

Notwithstanding this wasting away, and the certain signs of approaching death, this unhappy man persisted in his chimerical ideas; he was convinced that sponges, keys, and other foreign bodies were introduced into his stomach. In order to prevent his food touching his plate, he endeavoured to keep it suspended in the air. His anguish when it fell was frightful. The very day of his death he repeated that I had poisoned him, and that his pillow was filled with diamonds which he was accused of having stolen. He expired saying that the morsel of food which he was eating was poisoned.

The hallucinations of lypemaniacs are related to the cause and nature of their disease, the bent of their ideas, and the germ of their passions; thus they should be a more or less faithful reproduction of these origins. Those who have studied chemistry and physic, or who have heard much about these sciences, believe themselves pursued by physicians, electricity, or magnetism. Those who have been rich, who have lived by their own industry, who have met with pecuniary losses, imagine that some one wishes to rob them, or that the police are about to fetch them away. In a word, the hallucination itself in a majority of cases is a token which affords many useful hints.

Some melancholic victims of hallucination are anxious beyond description; they cannot conceive why one should apparently take every sort of precaution to prevent them killing themselves, and yet furtively point out to them the means of doing so. They are surly and repulsive in manner, despair is depicted in every feature; their visage is motionless, earthy, and yellow; their eyes hollow, cast down, and of the same tint, or extremely bloodshot. They are subject to headache, more or less acute, ordinarily in the frontal region, and particularly about the root of the nose. They experience beating in the head; they are tormented by sleeplessness, or if they sleep, are troubled by dreams and fantastic apparitions.

Sometimes patients are conscious of their delusions, but soon the impression reappears in all its force. We said one day to a man suffering from hallucination, who hesitated as to the reality of his sensations: "How can these people whom you have never seen, and do not know, whisper to you against your will?" He replied: "It is incomprehensible, I cannot explain it, but so it is." We insisted on the impossibility. "You do not believe," he answered, "because you have not seen; I have seen, and therefore I believe." This is the unassailable position of the hallucinated; they have seen, felt, tasted, touched, like other men. The most indifferent, as also the strangest and most opposite objects fall within their system of interpretation. One patient assured us that the writing and drawings of other patients upon the walls were signs of the sun and of the stars, and that he found the same characters on the pebbles in the garden. A naturalist, who had formerly made collections, and who continued to do so in his madness, collected stones, sand, and leaves, which were to him so many discoveries, respecting which he reckoned on publishing some day important memoirs. A lady lived under the conviction that she was the subject of general attention; each morning she brought us the newspaper, in which she read her own history in print.

The changes of persons and of things, and the interpretations consequent upon them, may give rise to a host of disagreeable scenes. One of our female boarders was one day travelling by railway; she thought she saw in her compartment a military man in an indecent posture; she changed her carriage, and then met with several men, who looked at her and addressed licentious conversation to her. She became excited, and was obliged to be brought back to our establishment.

This species of sensations does not exist solely among persons under hallucination. We meet with defiant, suspicious, impressionable people in the world, who attribute to others their petty passions; or exaggerating their own importance, imagine that they are the subject of remark and observation; they interpret in this sense a gesture, a movement, a look; whatever is said or done bears reference to them, and this system of interpretation has frequently resulted in offensive language, taunts, threats, and provocations. This turn of mind belongs to our national character, and the assertion is not without reason that one-half of the human species mocks the other half. Our object in showing these instances of the near approach of the analogies of reason and madness, is to develop some of our ideas respecting madness, and especially that which leads us to look upon this disease as a moral exaggeration, or, as we are tempted to call it, a caricature of the reasonable man. Madness, we wrote a few years ago, does not, as the world imagines, create a being afresh; in ordinary cases it exaggerates the qualities and defects of the subject, and taking away his mask, causes him to think and act aloud and openly; but as there is no such thing as an absolute theory, it may also completely change his character. The interpretations, more or less plausible, given by persons under hallucination of all that is said or done around them, in accordance with their illusions, has also its analogue in the passions; the logic of both reposes on the same base, the favourable interpretation of all which leads to the satisfaction of desires or the realization of fears.

Hallucinations and illusions are often related to actual facts. A magistrate was called upon to act in an affair in which one of his intimate friends was seriously inculpated; he determined to allow the accusation. On the eve of the day on which his report was to be filed, the wife of the accused entered his chamber in the middle of the night, and throwing herself at his feet, made all those appeals to his heart which the situation would give rise to; finding him immovable, she fainted and fell down, and was carried out dying. The magistrate did not fail in his duty, but this event took hold of his mind, and in the end engrossed all his thoughts; he fancied that he had overstepped his duty, and that every one reproached him; life became insupportable to him, and after several attempts at suicide he was brought to our establishment. There likewise he heard the patients, their relations, and the attendants reproach him for his conduct, and it became necessary to feed him mechanically. In this case, the hallucination was traceable to the exaggeration of a good disposition in an impressionable, timorous man: in the following example it was the cry of conscience. A physician, of feeble character, was tempted to lend his assistance towards procuring an abortion. The miserable man who seduced him, profiting by his position, afterwards compelled him to give him a sum of money. The physician was most deeply affected; he was attacked by monomania with stupor, remained immovable, and refused to eat. All who surrounded him appeared to him to know his secret; they called him opprobrious names, and incessantly told him that he would end his days on the scaffold. "Why should I live? I am dishonoured and lost," was his cry, and he died of consumption. Patients have often said to us—We are accused of theft; it is true we have abused the confidence that was placed in us! It is matter of observation, therefore, contrary to the opinion of some learned physicians, that hallucinations and illusions are sometimes one of the forms of remorse.

Under other circumstances a pecuniary loss among very economical individuals produces the hallucinations. A merchant suffers heavily from a failure. He becomes sad, and fancies he has lost everything. How can he take food when he cannot pay for it? In proof of his opinion he shows us his filthy linen and ragged dress; but both are neat and fitting. We attempt to prove this by comparison with other things, but he will not alter his opinion, and becomes angry if pressed. Then, it is the man of regular habits who quintuples his loss, and his madness is but the exaggerated consequence of his habitual ideas of economy. In the following observation, on the contrary, it is the character which is transformed. A young lady, gifted with a generous heart, loses a sum of money which she has lent. Voices tell her that she is ruined; her parents are the authors of her misfortune. She hides her wrong, imposes upon herself the hardest privations, her avarice is without bounds. They are phantoms which speak to her; and, above all, she sees an eye which watches over everything she does.

It has been said that delirious conceptions of the most *bizarre* character have their point of departure in an actual thought or sensation. Without generalizing this observation too much, verified nevertheless in many cases, we have demonstrated its correctness, both in hallucinations and illusions. An *hallucine*, who complained that his teeth (which were bad) were gnawed, said, "During the six years I have lived with you, they have prevented me thinking." Was not this the index of a confusion of ideas which he felt to escape him, and which he wished to lay hold of?

Although the belief in the reality of their false sensations is the characteristic sign of the hallucinated, some of them occasionally entertain doubts. A lunatic, who every instant saw figures change and objects assume another aspect, knew that he dreamed while awake. Another before whom passed cars filled with corpses, exclaimed in a despairing voice: "I know that when first ill these figures were hallucinations, but now they are realities!"

In general, the hallucinations and illusions in melancholic monomania have a very striking character of fixity and duration. It sometimes happens, however, that they will cease all at once, and when least expected. A lunatic would remain many days without eating, because a voice had forbidden him. This act, which during many months had been often repeated, had led to very marked wasting and enfeeblement, would cease suddenly because the voice ordered him to take food. It was then necessary to regulate his meals, or he would devour all he could seize upon. Another *halluciné*, who had been fed by force, but in a very insufficient manner, caused great uneasiness for his life. Esquirol, in consultation, looked upon the patient's position as serious, prescribed a suitable treatment, and particularly recommended that he should be removed from the asylum into the country. The same day I gave a dinner. It had occurred to me to place the lunatic at table. The sight of the different meats produced upon him a favourable impression; he ate with very great appetite, and from this time he never again refused his food. These sudden changes are also observed in ordinary life. A man will not give heed to any advice, all seems lost; a word from some one else, a sudden thought, quickly changes completely his disposition.

The intellectual locutions of mystics, which M. Baillarger denominates psychical hallucinations, and which resemble interior revelations, cause the hallucinated to believe that their thoughts are penetrated. Nothing is commoner than to hear them say—"Why ask me? You know all—you comprehend all!" Sometimes they explain this fact by the action of fluids. An *halluciné* was persuaded that there was an exchange of electricity between his body and that of his child, which enabled him to know all its interior dispositions. This insane hallucination is found among superstitious beliefs, and it is not necessary to seek illustrations elsewhere. There are many persons who are persuaded that by drawing cards they can divine thoughts and predict the future.

Divination of the thought, resting on the belief in good and bad spirits, is far from being extinct. It is to this belief that we should refer the throwing of lots, of which we have had five examples, one following another: the hallucinated attribute to this influence their visions and illusions. We should be wrong if we fancied that this error was exclusively found among the peasantry.

There is a very intelligent nation which admits the existence of *jettatores*, and this even among the elevated classes of society. When a *jettatore* is in sight, he is evaded, and if he comes directly towards an individual, the latter seeks to avert peril by making a horn above the forehead with a finger. This I have witnessed myself.

Sometimes the hallucinated, attacked with melancholic monomania, see appear in their visions persons who they know to have been dead some time. A lady saw the figures of her parents and friends of whom the death or absence extended back twenty years. Blake, the seer, evoked at will the celebrated personages with whom he wished to converse, and they prayed him to interrogate them. Apparitions of this class have been noted among opium-eaters. Thomas de Quincey relates in his *Confessions*, that he often saw, when awake, a multitude of ladies. "I heard them say to me," he states, "or I said to myself: 'These are the wives and daughters of those who meet together in peace, who sit at the same tables, and who were allied by marriage or by blood. These ladies danced and were as seductive as at the Court of George IV. Nevertheless I knew, even in my dream, that they had been buried nearly two ages.'"

THIRD CATEGORY.—*Illusions only.*

Of thirty cases of illusions alone, twenty-five were exclusively of a gloomy nature. There is nothing to distinguish them from hallucinations connected with those illusions concerning which we have just enumerated what appeared to us the most striking features. There are the same gloomy, threatening, terrifying impressions. We have only remarked pretty frequent illusions of touch in connexion with the sexual organs. One lady of sixty-eight, neither handsome nor well made, asserted that certain young persons violated her every night. Another complained to her husband that the devil, after harassing her for a long time with his importunities, had at last got the better of her, and was in the habit of exacting regularly the rights of a husband.

Fear of the devil, and a dread of future punishment used formerly to exercise an immense influence upon the intellect. Demonomania, which had become much less common since the eighteenth century, has, to a certain extent, reappeared with the return of religious sentiments, as though evil was an inevitable accompaniment of good. In six years we have noted fifteen cases at our establishment.

Doctor Macario is of opinion that this type of madness is common in provincial lunatic asylums; which he takes to be a proof that materialism has not struck the root so deeply in French soil as might be imagined. He points to a host of hallucinations and illusions among demonomaniacs. The devil presents himself to them under the shape of an animal, sometimes half-man, half-dog, or half toad, or as a flash of lightning. He enters the body, speaks through the mouth, takes possession of the faculties, pricks, burns, tears out the heart, the brain, the entrails, and torments in a thousand ways; he diffuses an unpleasant smell of brimstone or of a goat, &c.

At other times, and this peculiarity is observable mostly among females, the evil spirit holds obscene discourse. Some demonomaniacs are lifted into the air and carried off to the infernal regions, where, struck with fright and horror, they look upon the tortures of the damned. Others fancy themselves transformed into animals, trees, or fruits, or reduced to ashes, and then, like another phœnix, brought to life and regenerated. Some are surrounded with hideous reptiles or with corpses: some fancy they have sold their souls to the devil, and signed the contract in blood; they believe themselves lost for ever. There are others who are never to die; at the end of the world they will be alone upon the earth. Some are more fortunate: the devil protects them, teaches them the secret of making gold, prophesies to them future events, unveils for them the mysteries of hell, and grants them the power of working miracles; at their bidding lightnings flash, thunders roll, the earth yawns, and the dead come to life.

Madame C., a lady of foreign birth, about forty-eight years of age, was always lively, impressionable, and enthusiastic. Brought up in the midst of the most superstitious practices, without education, as is the custom of her country, she has had for six years an intermittent melancholy, which, after many attacks, presented itself under a new aspect. This lady, who for a long time had neglected the duties of religion, was attacked by remorse: she thought herself damned. Harassed by this idea, she abstained from any kind of food for several days. When brought to my establishment, she had at times furious attacks. At our first interview, she pronounced with volubility these words: "In Hell, damned; you in Paradise" Quiet at first, she began to call out, complaining that she saw devils, that she was surrounded with flames. "I am lost—my children are lost—save me!" Exclaiming thus, she shrieked madly, beat her head against the walls, broke the windows, and tore her clothes. Every minute she would ask for drink, as though burnt by an internal fire.

For three days she was quiet, and then the same ideas returned. Her bristling hair, her haggard eyes, and her prolonged srieks, made her a striking representation of one possessed. From her mouth escaped an abundant mucous discharge, which she spat occasionally into the faces of the assistants. The fear and sorrow stamped upon her features were but too sure indications of the effect of her terrible visions. When the strait-waistcoat was removed, she would bruise her breast with the heavy blows she struck upon it. Frequently she tried to dash her head against the walls.

During the last month of her illness, her cries became so continuous that she was obliged to be removed to a distant room. There, always huddled up, her face blue from the bruises she was constantly inflicting upon herself, her eyes staring, sunk in their sockets, and bloodshot, her skin cadaverous, yellow, and wrinkled, her aspect threatening, her voice hoarse through her cries that she was lost, damned, that the devil was in her body, that he tortured her, and prevented her shutting her eyes by his continual apparitions, she presented all the symptoms of the most hideous despair. She was continually begging her attendant to save her, and to deliver her from her fate.

This frightful nightmare of course disturbed all her functions. Thus, she refused food at an early stage. She would go for three or four days without taking anything. At the last period of her existence she was fifteen days without eating, taking only occasionally a cup of coffee. This she would often decline, saying that it burnt her; that it had a disgusting taste, which was caused by the really unbearable foulness of her own breath; and her feverish state forbid forced nourishment.

Before long the eyes and the nostrils were filled with purulent mucus, announcing a fatal termination. Toward the end of her life she afforded an instance of the effect which diseases of the nervous system may have upon the physical organization. Reduced to the last stage of marasmus, having given up food for some time, she had twisted her limbs one within another, and had heaped herself up together with such strength, that all attempts to bring her to a natural attitude were unavailing. She expired, preserving the same rigidity, a prey to the same hallucinations, refusing drink, and repeating continually that she would not die.

We have spoken of the lunatics who imagined that the devil was in them, and that they were surrounded by flames, and of others who thought that he made grimaces at them, and threatened them. One young lady used to follow us about continually, to tell us that the human face was lost—that we were all devils; for illusions frequently in this case are added to hallucinations.

Among women, the apparitions of the devil are connected with sexual periods, which is explained by the symptoms of hysteria, erotomania, and nymphomania which are so common with the female sex, and which so abound in all the works of writers on demonology.

According to those authors who have treated of these matters, the object of Satan being to instigate the greatest crimes, he changes himself into a man when dealing with women, into a woman when dealing with men. It is the incubi who have to do with women, succubi with men.* Cœlius Aurelianus tells us, on the testimony of Salimaque, the partisan of the Hippocratic doctrines, that an infectious type of incubus had appeared at Rome, and that many persons died of it.†

In our days, cohabitation with the devil is much less common than it formerly was. Among the hundreds of lunatics who have come under our observation, we have only collected one or two authenticated cases. Hallucinations of this kind have more specially for their object angels, or men clothed in imaginary charms, or frequently the superintendents of asylums.

However, M. Macario has reported several cases of cohabitation with demons.

The facts collected by M. Macario and by ourselves show that there do exist lunatics who imagine that they have sexual intercourse with the devil: but, in the greater number of cases, the fantastic shapes assume the human form.

Madame B—— is persuaded that she is about to marry a man, noble and powerful, who has possession of all her sympathies. Prepossessed with this idea, she pays no attention to her real husband. Every night, she tells me, she is visited by the Angel Raphael, a handsome fair young man, clad in black, with pale face, who converses with her in the most gracious manner; her mattress, too, shakes violently, so that one would imagine a man was inside.

Mdlle. Z——, seventeen years of age, was brought to our establishment for a mental affliction caused by love. The first symptoms broke out three days ago. Her face reveals infatuation and happiness. Her lover does not leave her, he follows her everywhere, he lavishes upon her the most endearing names: when he retires, she throws herself on her knees, begs pardon of him, and prays him not to reduce her to despair. She sees him in the clouds, crowned with roses; he bestows upon her the sweetest smiles.

It is a most melting scene when she sings to her lover the song of the "Mad Girl." The interest she excites is such that old patients who have been ten years in the house, cluster round and listen to her with manifest delight. Never has the part of Nina been rendered with more truthfulness or ease. It is the only instance for more than twenty years in which I have seen a case of amorous

---

* J. Garrinet, *Histoire de la Magie en France.* Paris: 1816.

† Cœlius Aurelianus, *Chronic morb.* bio. i. cap iii. De Incubone. Lyon, 1567. Horst, *Dämonomanie oder Geschichte des Glaubens au Zauberei und dämonische Wunder mit besonders Berücksichtigung des Hexenprozesses seit dem Innocentius* VIII. Frankf. 1828. Friedrich's *Litterargeschichte der Peth. und Ther. der psych. Krankheiten*, p. 127.

madness which might serve as a model for the stage. The symptoms which almost always accompany this form of madness render an exact imitation of it impossible.

To show his love, her lover brings her bouquets of flowers, and makes her breathe the most delicious perfumes. "See these roses," she cries, "they scent the air, the room is filled by them." Her speech, her looks have no freedom, they are addressed to the same person. Thus this madness is an object of study. The concentration of Mdlle. Z——'s thoughts is such that one can scarcely get from her even a few words. The ecstasy calmed rapidly; she heard still the voice of him she loved ; but soon reason return, her hallucinations ceased; and after eight days of seclusion all the symptoms had disappeared.

Hallucinations or illusions of touch are observed in other cases. One patient, of the melancholy type, could not sufficiently express his indignation at the horrible manœuvres to which he was subjected. Sometimes it was a woman who got into his room under cover of darkness, and provoked him in all sorts of ways; sometimes it was men who abused him. He explained his passive state by the manœuvres of his persecutors who put him into a state of somnambulism.

Illusions of touch are often very painful. We have given instances of patients subject to hallucinations who had a conviction that they were being beaten severely. Frequently we have been struck by the apparent truthfulness with which they told their wrongs. One old lady for years persisted in accusing some evil-disposed persons of throwing upon her some deleterious substance, and of reducing her to ashes. Another, under the influence of these sensations, kept crying murder! with all her strength. Another lady assured us that every night persons fastened her to the ground for the purpose of torturing her ; that for three years she had been unable to sleep. The parts which were the seat of this ill-usage bore scars of old burns.

Alterations of faces and objects were observed among most of the thirty patients. One case of illusion of sight which deserves special mention is that of a man, aged forty, who since he was thirteen years old had seen copper spots upon his hands; he was every instant washing his hands to remove the spots, to avoid poisoning those who lived with him. For twenty-seven years no one had any knowledge of this delusion, which he made known to one of his relations for the first time the day he entered my establishment. Like many melancholic monomaniacs, the individuals who were a prey to illusions alone asserted that they were made to breathe a tainted atmosphere, and eat decayed food. All these facts having been already noted, we will not stay longer upon the subject.

*General Sensitiveness*—Illusions are not produced by a special sensitiveness only; they may also spring from a general sensitiveness. In hypochondriacal monomania especially, illusions produced by disease in some of the organs of the body are most frequently met with; the nervous state which takes possession of the system is very favourable to their production. We have collected a number of instances in cases of melancholic monomania, but we shall limit ourselves to a few quotations. A lady who was suffering from prolapsus uteri declared that she had an animal in her inside, and begged for scissors or some instrument to cut it out. Another patient, who had bad teeth, complained constantly, and frequently with threats, that I inserted some deleterious substances into his mouth to cause them to decay and spoil them. Another female patient said she saw a cloud of plaster-dust fall, which wounded and choked her. Very frequently rheumatic pains, neuralgia, and, in some instances, old scars of burns, cause lunatics to imagine that they are being beaten, burnt, or tortured. A somewhat common illusion among women is to fancy themselves pregnant. One old lady of seventy used to give out every year about the same time, that she was with child. She felt the movements of the child, got together her baby-linen, and lay in, but never troubled herself about the offspring.

Among the interesting facts we have met with in the study of the hallucinations and illusions of melancholic monomania, we give the following: A lady who was troubled with hallucinations, which are a useful study in their connexion with psychology, experienced suddenly a morbid acuteness of sight which lasted several hours. She could see quite distinctly at a considerable distance objects which were to us very confused; she declared even that she could see stars which were invisible to us. In order to prove the correctness of this state, we showed her a certain vessel, which was placed at a great distance, so that it was impossible to distinguish its shape, but which we knew was still there; she pointed it out to us immediately. This phenomenon vanished as it had appeared.

It is evident that this acute condition of the senses or of general sensitiveness is, in a great number of cases, the result of a morbid state of things; though we must not forget that under other circumstances it depends upon habit and exercise. Many and many a time we have known in the course of a disease the ear distinguish sounds which escaped others, yet became very painful to the patient; the nose perceive odours of which none of the assistants were conscious, and which arose from substances almost microscopic and scentless. A patient afflicted with a gastralgic affection is confined to his bed for twenty days without sleep or nourishment. At the end of that time he sits upright, and is astonished to find himself as strong and active as he was before his illness. Utterly astounded, he attempts to leave the bed, and feeling no weakness, he gets up, takes a step or two, and then a walk; and finds his strength quite returned. He examines his effects, his books, puts in order what had been disturbed. The comparison between his present state and his past sufferings, and the recollection of his treatment disquiet him. Three quarters of an hour pass, and no change in his condition; when all of a sudden everything appears to turn round him, and he has only just time to throw himself upon his bed. Convalescence was only commencing, and required some time to establish itself completely.

### 4. *Of Hallucinations in Cases of Monomania.*

Monomaniacs, like other lunatics, are subject to hallucinations and illusions; frequently, indeed, hallucinations and illusions are the sole characteristics of their madness, and are the cause of the perversion of their affections and the disorder of their actions. (Esquirol.) These false impressions are the reflection of their inclinations, tastes, hobbies, feelings, &c. Gods, kings, grand people, men of genius or of inspiration, civilizers or regenerators of the world, creators of new religions, all present, in the hallucinations connected with their mad conceptions, those characteristics of exaggeration, self-conceit, changeableness with respect to shapes, absurdity, childishness, and confusion which we have shown to exist in melancholic monomania. M. Lélut's patient, whose case we have reported, and whose intention is directed by the voice of God to propagate religious ideas, is carried away by them, instead of nursing and developing them. The madness is patent; thus God bids him lie down; his stomach speaks to him. The civilizer of the world spoken of by Leuret in his fragments, after having sought to better the condition of mankind in the most opposite countries, finishes by publishing a ridiculous pamphlet entitled, *Humanisation.* Noël, to whom Dr. Cazauvieilh has devoted a long article, wishes to bring mankind back to unity of faith. His speeches are those of a believer, of a person who has one fixed idea of a certain weight; he has heard for fifteen years a celestial voice which urges him to shun all society, that he may withdraw himself from the universal perdition which reigns therein. But this man, animated as he is by generous sentiments, throws aside his clothes and keeps only such as are strictly required by decency. He works two miracles; first, he opens a closed door by touching it with his finger; and secondly, he dies, and comes to life again after a few hours. This same individual gives orders, but will never obey; he gets angry and furious when his will is resisted. None but his adepts are pure enough to approach him. Noël appears at last to come back to practical ideas; he keeps a school, but it is clear that he has not lost his old delusions, such as his "calling, unknown of men," "his prophetic mission," "the approaching end of the world." At last, though preserving still these ideas, he takes again to his old trade as joiner.* All the other cases that we have analysed have shown us the same contradictions, the same puerilities, in a word, the same symptoms of madness.

In the twenty-nine cases of the hallucinations of monomania which we have collected, gathered from among individuals who were constantly under our observation, and whose madness sprung from pride, vanity, mysticism, love, jealousy, &c., the hallucinations and illusions were characterized by apparitions of virgins and angels, by ventriloquism, by figures of naked women or fantastic shapes. Extravagant ideas were suggested to the patients, dangerous orders given them: they were subjected to magnetic influences. Independently of these visions and conceptions, many of those who were subject to hallucinations saw faces and objects change shape. A comedy was acted round them; and, as sorrow appears as frequently in all the different forms of madness as in all the circumstances of life, many of them heard threatening voices, saw evil beings, breathed in imagination tainted air; and, in fine, there was found in a good number of these cases of monomania the common type of the hallucinations and illusions of mania and of melancholic monomania.

From the examination which we have undertaken we do not hesitate to declare that none of the cases which are our special study have presented the rational systematization which flows from intelligence and education, and which can alone lead to fruitful results. All the hallucinations of these patients were imprinted with the stigma of madness, whether studied in respect of their causes or manifestations, or followed in their progress, changes, or termination.

### 5.—*Hallucinations and Illusions in Puerperal Insanity.*

The insanity of pregnant women, of newly-delivered women, or of nursing women, should, it appears to us, follow immediately upon the different phases we have just studied, because it appears chiefly under two of those phases—the maniacal and, more especially, the melancholic (lypemaniacal), though it may be observed also in some instances in a monomaniacal, or stupid state. Dr. Marcé, in the useful treatise which he has published upon

* Cazauvieilh, *Du Suicide et de l'Aliénation Mentale*, p. 158. Paris: 1840.

this subject,* while establishing the existence of hallucinations and illusions, says that they have no special feature. Out of twenty-eight cases of puerperal insanity which we have noted, in eighteen there were hallucinations and illusions, mostly of a sad or terrible nature, since melancholy predominated; in one case of stupidity there existed disorders analogous to those described by M. Baillarger. To sum up, we have met, in this form of insanity, the general characteristics pointed out in the descriptions of these phenomena among maniacs and in melancholic monomania; so that they do not require any special remark. Owing to the predominance of the melancholic type, we cannot be surprised to find a tendency to suicide occasionally manifesting itself; it existed in three of our cases. Out of 111 cases of puerperal insanity, collected at Bethlem and published by Dr. J. Webster, a tendency to suicide was observed in thirty-two.

### 6.—*Hallucinations and Illusions in Two-phased Insanity (folie à double forme).*

This variety of insanity, which has been described under its two aspects in the general course of the disease, has been made the subject of more special study by MM. Baillarger and Falret. The first has named it "Two-phased Insanity:" the second "Circular Insanity." Many of the cases that we have observed showed very marked symptoms of mania and lypemania. Out of the twenty-seven cases which figured in the list, hallucinations and illusions were observed in fourteen: they were characterized by the peculiarities proper to the two forms of insanity which they resembled most commonly; and it is on account of this double aspect that we have placed them, like puerperal insanity, next in order to mania and lypemania. The false sensorial perceptions having offered nothing which was special, or which would not come under one of the two great heads already spoken of, we have not thought it necessary to enlarge upon this variety.

*Recapitulation.*—Hallucinations affect in preference the monomaniacal form: they are also more easily recognised in this kind of delirium.

The hallucinations and illusions of monomania have special characters of fixedness, intensity, tenacity, and expression. They are usually of a melancholic nature in lypemania, and present every shade of grief and despair, but they change frequently with respect to depth, and are in this respect very mobile.

Hallucinations are frequently the reflex of the idea prevailing at the time.

Hallucinations alone are numerous, but less so than hallucina-

---

* L. V. Marcé, *Traité de la Folie des femmes enceintes, des nouvelles accouchées, et des nourrices,* &c. Paris: 1858.

tions and illusions combined. Illusions alone form the shortest catalogue.

Hallucinations are exaggerated, false, absurd, having no connexion with cause, either directly or by an exaggerated relation, confused and changeable in their external manifestations.

Nothing is more common in this kind of hallucination than to observe changes of persons and objects; so that the patients live in a fantastic world. These transformations are interpreted by them in the most ingenious manner, and in the way most favourable to their delirious conceptions; and they yield to no explanation.

Hallucinations frequently arise from actual causes exaggerated or perverted by disease.

Patients who have had a relapse sometimes admit that their visions in former illnesses were false, but are real in their present state.

Hallucinations may vary, change, become intermittent, or disappear.

Patients who are subject to psychical hallucinations believe sometimes that others know their thoughts.

Women frequently experience sexual illusions.

Hallucinations and illusions of smell and taste are generally of an unpleasant nature.

Anæsthesia is often observed in persons subject to melancholic hallucinations. Hyperæsthesia has been remarked in some cases.

General sensitiveness may give rise to hallucinations and illusions.

---

